# Aligned nanofiber scaffolds improve functionality of cardiomyocytes differentiated from human induced pluripotent stem cell-derived cardiac progenitor cells

**DOI:** 10.1038/s41598-020-70547-4

**Published:** 2020-08-11

**Authors:** Mei Ding, Henrik Andersson, Sofia Martinsson, Alan Sabirsh, Anna Jonebring, Qing-Dong Wang, Alleyn T. Plowright, Lauren Drowley

**Affiliations:** 1 Discovery Biology, Discovery Sciences, R&D, AstraZeneca, Gothenburg, Sweden; 2 Bioscience Cardiovascular, Research and Early Development, Cardiovascular, Renal and Metabolism (CVRM), BioPharmaceuticals R&D, AstraZeneca, Gothenburg, Sweden; 3 Advanced Drug Delivery, Pharmaceutical Sciences, R&D, AstraZeneca, Gothenburg, Sweden; 4 Medicinal Chemistry, Research and Early Development, Cardiovascular, Renal and Metabolism (CVRM), BioPharmaceuticals R&D, AstraZeneca, Gothenburg, Sweden

**Keywords:** Drug discovery, Stem cells

## Abstract

Cardiac progenitor cells (CPCs), capable of differentiating into multiple cardiac cell types including cardiomyocytes (CMs), endothelial cells, and smooth muscle cells, are promising candidates for cardiac repair/regeneration. In vitro model systems where cells are grown in a more in vivo-like environment, such as 3D cultures, have been shown to be more predictive than 2D culture for studying cell biology and disease pathophysiology. In this report, we focused on using Wnt inhibitors to study the differentiation of human iPSC-CPCs under 2D or 3D culture conditions by measuring marker protein and gene expression as well as intracellular Ca^2+^ oscillation. Our results show that the 3D culture with aligned nanofiber scaffolds, mimicing the architecture of the extracellular matrix of the heart, improve the differentiation of iPSC-CPCs to functional cardiomyocytes induced by Wnt inhibition, as shown with increased number of cardiac Troponin T (cTnT)-positive cells and synchronized intracellular Ca^2+^ oscillation. In addition, we studied if 3D nanofiber culture can be used as an in vitro model for compound screening by testing a number of other differentiation factors including a ALK5 inhibitor and inhibitors of BMP signaling. This work highlights the importance of using a more relevant in vitro model and measuring not only the expression of marker proteins but also the functional readout in a screen in order to identify the best compounds and to investigate the resulting biology.

## Introduction

Heart failure after myocardial infarction is a clinical condition that causes high morbidity and mortality^1^. The only currently available curative treatment of end-stage heart failure is heart transplantation, whereas other treatment options merely slow the progression of the disease. Therefore, there is a large unmet medical need for treating heart failure that is driven by the loss of functional cardiomyocytes (CMs) which occurs during myocardial infarction^2^. Thus, cardiac regeneration is now seen as an attractive strategy for repairing damaged heart tissue and for treating heart failure^2–4^. Recent studies have demonstrated that transplantation of human embryonic stem cell (ESC)- or induced pluripotent stem cell (iPSC)-derived cardiac progenitor cells (CPCs), CMs, or cardiac muscle patches, formed new functional myocardium and improved cardiac function in rodents, swine, non-human primates, and in humans^5–9^. Therefore, CPCs are very promising candidates for improving the function of dysfunctional myocardium, and for the identification of molecules increasing differentiation of CPCs to CMs which could enhance the therapeutic value of these cells for heart failure.

CPCs can be generated from iPSCs and we have previously demonstrated the multi-lineage differentiation capacity of iPSC-derived CPCs into different cardiac lineages: CMs, smooth muscle cells (SMCs) and endothelial cells (ECs)^10^. Screening of a diverse compound library using human iPSC-CPCs showed that XAV939 (an inhibitor of Wnt pathway signaling), Dorsomorphin (DM) (an inhibitor of AMP-activated kinase (AMPK) and BMP signaling), RepSox (an inhibitor of TGF-b type 1 receptor ALK5) or other structurally diverse inhibitors of ALK5^10^ promote cardiac differentiation of human iPSC-CPCs. This study demonstrated that the human iPSC-CPC assay can be run in a medium to high-throughput phenotypic screening approach to identify novel and potent regulators of cardiac regeneration and potentially yield new drugs or new therapeutic mechanisms for treating heart failure.

Our previous iPSC-CPC differentiation study was performed in standard two-dimensional (2D) culture. It is well known that cells cultured on 2D flat glass or plastic planar surfaces often show different morphology and function compared to cells in the native tissue^11^*.* Many examples across different cell types have shown that cell morphology, function, and fate are influenced by the physical interactions of cells with the extracellular matrix (ECM)^12–16^. During the past 20 years, increased efforts have been made to culture cells in a more in vivo-like environment by using three-dimensional (3D) culture systems with relevant matrix components. Numerous studies have demonstrated that in vitro cellular models with cells grown in 3D culture, which structurally mimic the architecture of the ECM of the native tissue, have higher predictivity in in vitro models than 2D culture models for studying cell biology and disease pathophysiology, and for identifying therapeutic agents^17–19^. For example, HepG2 liver cancer cells in 3D spheroids produce their own ECM and are highly organized and ‘tissue-like’^20^, fibroblasts cultured with collagen gels and fibronectin-containing matrices that mimic in vivo 3D environment exert different drug responsiveness compared to cells growing in 2D cultures^21^. However, using 3D culture models, such as spheroids, organoids and organ-on-a-chip in drug discovery for screening large numbers of compounds (e.g. in a phenotypic screening campaign) can still be very challenging as these more complex assays are difficult to adapt to plate-based medium-to-high throughput screening and automation. In recent years, a number of plate-based 3D culture models, such as low attachment and hanging drop plates for spheroids, plates with nanofibrous scaffolds composed of electrospun synthetic nanofibers, and plates coated with hydrogels, have become commercially available. Such 3D cultures have been investigated in different cellular models to improve physiological relevance, including human adipose-derived stem cells^15^, rat hippocampal embryonic neurons^22^, bovine pulmonary artery smooth muscle cells^23^, and hepatocytes^24,25^. Adult CMs in vivo have an elongated morphology, but, CMs cultured in standard 2D condition do not show elongated morphology. Numerous studies have shown that aligned nanofiber scaffolds guide CM cell alignment along the direction of fiber orientation, promote adaptation of an elongated CM morphology, and improve CM function and maturation when CMs are cultured in 3D aligned nanofiber scaffolds^26–32^. The potential of engineered cardiac tissue like constructs obtained by seeding CMs on aligned nanofibers or into a 3D fibrin scaffold for treating myocardial infarction has been demonstrated^8,32^. In addition, several groups also reported that nanofiber scaffolds enhance cardiac differentiation of stem cells or progenitor cells^33–37^. Thus, there is a clear rationale to investigate if nanofiber scaffolds can improve iPCS-CPCs differentiation into CMs to provide a more effective and relevant model or phenotypic assay, particularly if scaleable for medium-to-high throughput drug discovery.

For this study, 384-well 3D nanofiber plates with aligned polycaprolactone (PCL) nanofiber scaffolds, which structurally mimic the architecture of CMs in the heart^38^, was chosen to investigate the effect of 3D scaffolds on human iPSC-CPC cardiac differentiation triggered by Wnt inhibition that is reported to promote iPSC-CPC cardiac differentiation. Protein and gene expression of CM and SMC markers, and intracellular Ca^2+^ oscillation were used for cardiac differentiation assessment. In addition, we studied if 3D nanofiber culture can be used as an in vitro model for compound screening by testing alternative molecules which have been shown to differentiate iPSC-CPCs in 2D culture.

## Results

### Wnt signaling inhibition induced differentiation of human iPSC-CPCs in 3D and 2D culture

**Figure 1:**
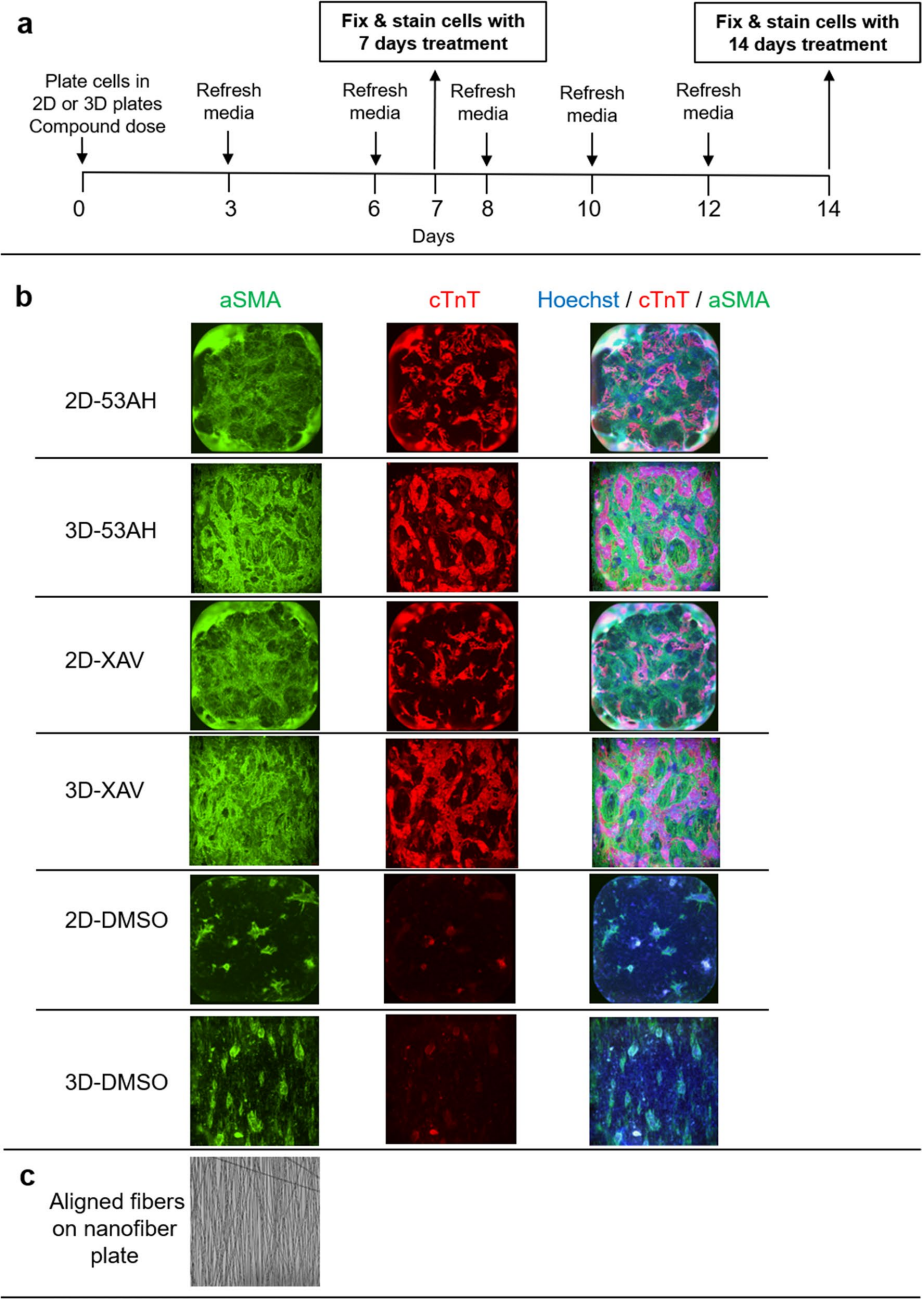
Schematic outline of the CPC differentiation protocol and immunofluorescence staining of CPC differentiation in 3D aligned nanofiber plates and 2D plates. (**a**) Outline of the human iPSC-CPC differentiation protocol: Human iPSC-CPCs were plated in 3D aligned nanofiber or 2D plates with the addition of compounds and DMSO control from day 0 to day 3, followed by culture in assay medium until day 7 or day 14 of differentiation. (**b**) Representative images of cells treated with 1.1 µM 53AH, 10 µM XAV939, or DMSO control at day 14 of differentiation indicate that human iPSC-CPCs are competent for differentiation to cardiomyocytes (express cardiomyocyte marker cTnT) and cells expressing αSMA. (**c**) A phase contrast image of aligned nanofiber plate. These images were acquired at × 4 magnification.

Human iPSC-CPCs were treated with 10 µM XAV939, 1.1 µM 53AH (a structurally diverse inhibitor of Wnt signalling), or DMSO control in triplicates. Cells were fixed at day 7 or day 14 of differentiation, then stained for cardiac Troponin T (cTnT) and smooth muscle actin (αSMA) for studying iPSC-CPC differentiation. These concentrations and time points were selected based on our previously obtained knowledge for the differentiation of CPCs to CMs and that described in the literature^10,39^. Figure 1 shows an outline of the human iPSC-CPC differentiation protocol, and double immunostaining with cTnT and αSMA of cells at day 14 in 3D versus 2D culture treated with XAV939, 53AH or DMSO, as well as aligned fibers on 3D nanofiber plates.

**Figure 2:**
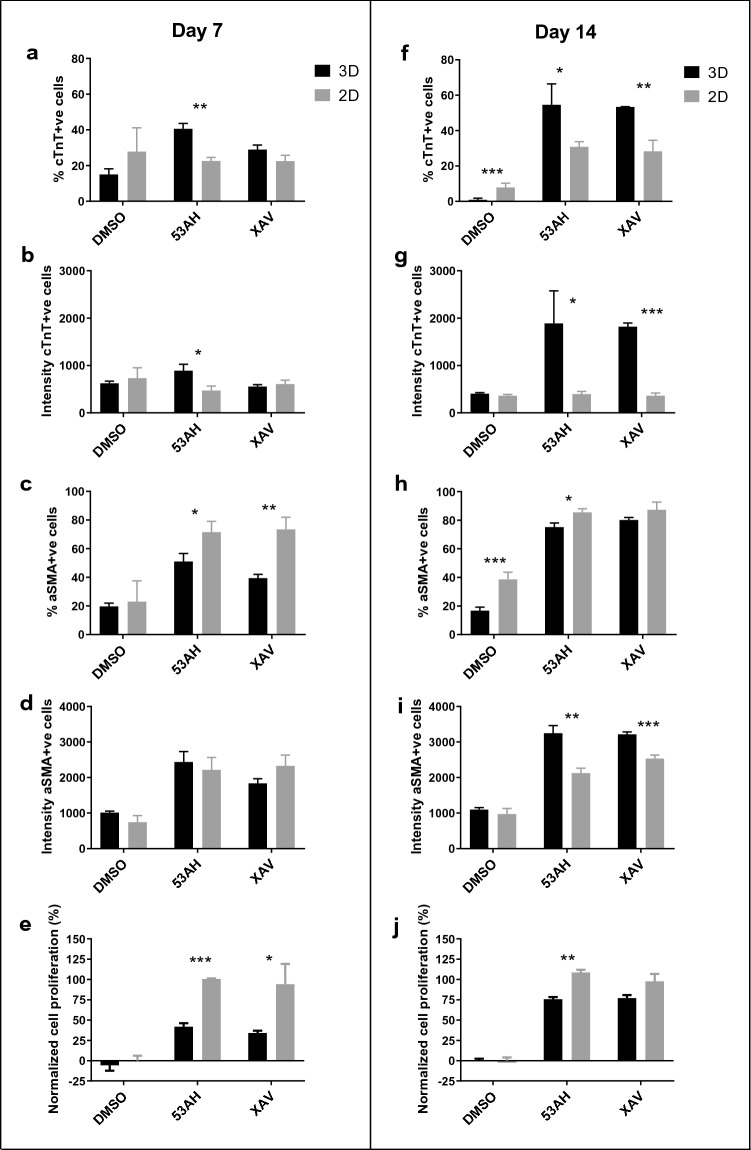
Effect of Wnt signaling inhibition on CPC differentiation and cell proliferation in 3D vs 2D culture. Human iPSC-CPCs were plated in 3D aligned nanofiber and 2D plates with the addition of DMSO control, 1.1 µM 53AH, 10 µM XAV939 from day 0 to day 3, followed by culture in assay medium until day 7 or day 14 of differentiation. The expression of cTnT and αSMA were studied by acquiring images at × 20 magnification. (**a**–**e**) At day 7 of differentiation in 2D and 3D cultures, % cTnT-positive cells, cTnT intensity of cTnT-positive cells, % of αSMA-positive cells, αSMA intensity of αSMA-positive cells, and effect on cell proliferation in 2D and 3D cultures. (**f**–**j**) At day 14 of differentiation, data of % cTnT-positive cells, cTnT intensity of cTnT-positive cells, % of αSMA-positive cells, αSMA intensity of αSMA-positive cells, and effect on cell proliferation. The effect on cell proliferation was normalized as the percentage of cell proliferation based on the on-plate DMSO controls. Results are presented as mean ± SEM. n = 3. *p < 0.05, **p < 0.01, ***p < 0.001 indicate significant differences between 3D and 2D cultures.

To quantify protein expression levels, automated analysis was performed using Columbus image analysis software (Perkin Elmer). Nuclear and cytoplasmic regions were segmented using images of Hoechst and combined images from cTnT and αSMA, respectively. cTnT and αSMA intensities were calculated, and cells were classified as cTnT-positive and/or αSMA-positive. At day 7 of differentiation, 53AH treatment resulted in a significantly higher fraction of cTnT-positive cells and higher cTnT intensity in 3D nanofiber culture compared to the 2D cultures (Fig. 2a,b). As the differentiation time increased from 7 to 14 days, the percentage and cTnT intensity of cTnT-positive cells in 53AH and XAV939 treated cells in 3D culture were increased (Fig. 2f,g).

The percentage of αSMA-positive cells was higher in 2D culture treated with 53AH or XAV939 at day 7 (Fig. 2c), as well as in 2D culture treated with 53AH or DMSO at day 14, compared to the corresponding 3D cultures (Fig. 2h). Higher αSMA intensity was also observed in αSMA-positive cells in 3D culture treated with 53AH and XAV939 at day 14 (Fig. 2i). No significant differences were found for other treatments at day 7 or 14 between 3D and 2D culture. Treatment with 53AH and XAV939 also increased αSMA intensity in 3D culture as the differentiation time increased from 7 to 14 days (Fig. 2d,i).

Our results also show that 53AH and XAV939 treatments induced significant cell proliferation in 2D culture at day 7 compared to the DMSO treatment (Fig. 2e). As the differentiation time increased to day 14 (Fig. 2j), the cell numbers were maintained at a similar level as seen at day 7, indicating that cell proliferation occurred mainly during the period of day 0 to day 7, and cells did not proliferate significantly between day 7 and 14. However, in 3D nanofiber plates, compound treatments induced less cell proliferation with only 34–42% proliferation at day 7 and 76% proliferation at day 14, as compared to around 100% cell proliferation in 2D culture at day 7 and 14. These results indicate that cells proliferate less in 3D nanofiber culture compared with standard 2D culture.

**Figure 3:**
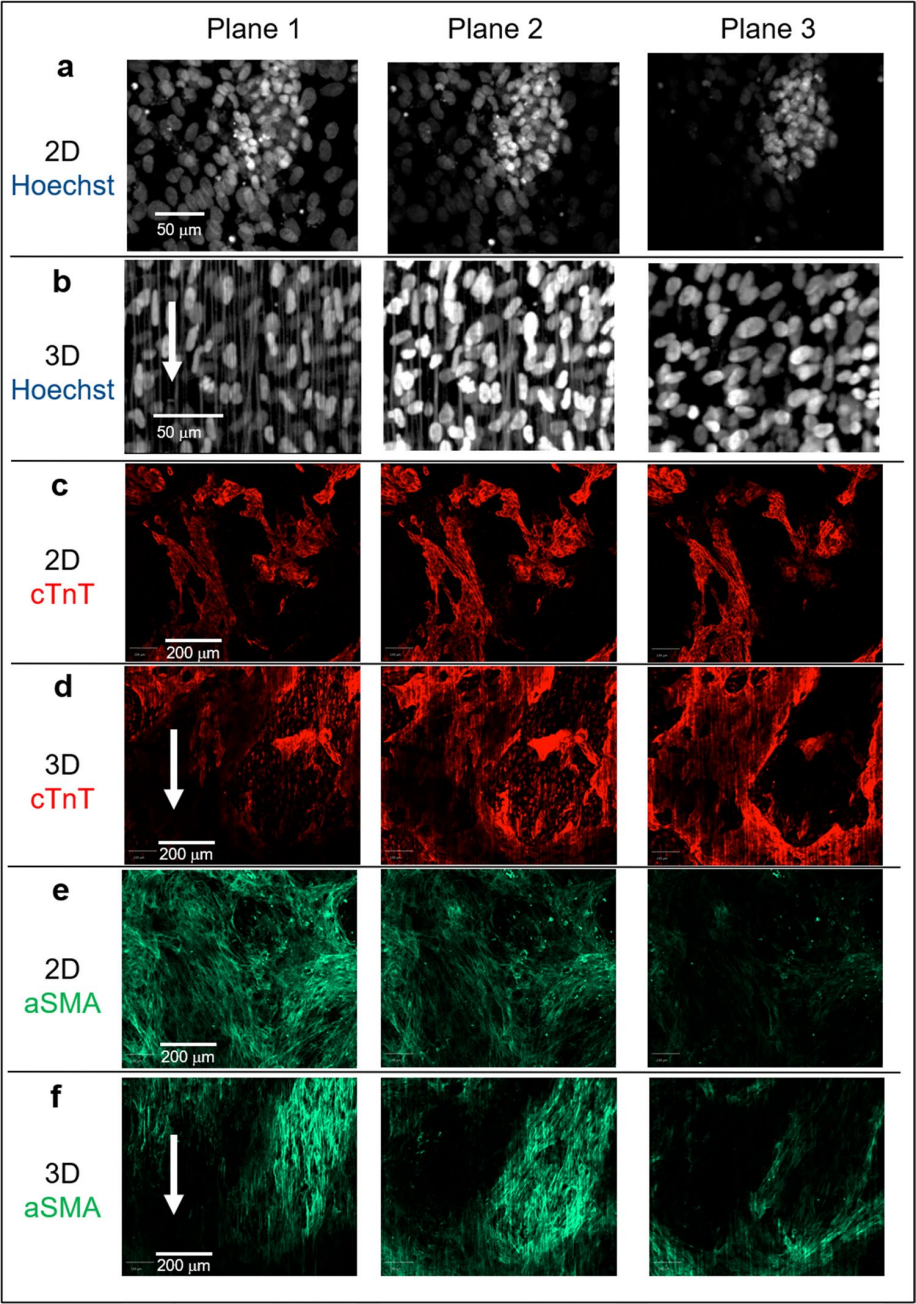
Confocal microscopy of CPC differentiation treated with XAV939 in 3D vs 2D culture. Human iPSC-CPCs were plated in 3D aligned nanofiber and 2D plates with the addition of 10 µM XAV939 from day 0 to day 3, followed by culture in assay medium until day 14 of differentiation. Hoechst staining (gray color), and expression of cTnT (red color) and αSMA (green color) were studied by acquiring images at 3 z-planes using × 20 air objective at 6 µm intervals. Plane 1 is close to the plate bottom level, Plane 2 is 6 µm above plane 1, and Plane 3 is 12 µm above plane 1. (**a**–**f**) Representative images of cells treated with 10 µM XAV939. Cells located closer to the fibers in 3D aligned nanofiber culture displayed elongated nuclei aligned to the orientation of the nanofibers (plane 1 in panel **b**). cTnT-positive CMs (panel **d**) were found to be located relatively distant from the fibers compared with αSMA-positive cells (panel **f**). Arrows have been added to plane 1 panel (**b**), (**d**) and (**f**) to highlight the fibers orientation.

Adult CMs in the heart have an elongated morphology. Studies from several groups have shown that aligned nanofiber scaffolds produced in their labs guide CM alignment along the fiber orientation and promote adaptation of an elongated CM morphology^26–28^. Nanofiber scaffolds in the 384-well aligned nanofiber plates from Nanofiber Solutions are relatively thin with a thickness of ~ 20 µm. Our nanofiber plate validation results show that human iPSC-CMs seeded directly on 3D aligned nanofiber plates and cultured for 10 days had an elongated morphology resembling native CMs in the heart, which is consistent with the data reported previously although the nanofiber scaffolds are thinner than those others have used (Fig. S1 in Supplementary Information). Conversely, CMs seeded in 2D culture did not elongate or orientate in any particular direction. Interestingly, CMs differentiated from iPSC-CPCs on 3D aligned nanofibers did not show elongated morphology as shown in Fig. 1. To better understand morphology of cells differentiated from iPSC-CPCs, the 3D and 2D plates with cells from day 14 of differentiation were imaged confocally at 3 z-planes using 20 × objective at 6 µm intervals. The confocal images show that many αSMA-positive cells located closer to the fibers in XAV939 treated 3D culture tended to display elongated nuclei aligned to the orientation of the nanofibers (plane 1 in panel b and f/Fig. 3), while cTnT-positive cells did not show a clear alignment with the fibers (plane 1, 2, 3 in panel d/Fig. 3). Cells in 2D culture plates did not show nuclei or cell morphology alignment (plane a, c, e in Fig. 3). The analysis of Width to Length ratio (WtL) shows that nucleus WtL ratio for cells treated with XAV939 in 2D culture were 0.54 ± 0.01 for plane 1, 0.55 ± 0.01 for plane 2, 0.56 ± 0.01 for plane 3, respectively, indicating no significant WtL ratio difference between planes in 2D culture. While nucleus WtL ratio for cells treated with 10 µM XAV939 in 3D culture were 0.47 ± 0.03 for plane 1, 0.54 ± 0.02 for plane 2, 0.58 ± 0.01 for plane 3. The lower WtL ratio at plane 1 in 3D suggests that nuclei at plane 1 in 3D culture were more elongated when comparing with nuclei upwards in the same sample, or comparing with nuclei on the same plane in 2D culture. Several reports have shown that nuclear morphology can be used as a surrogate for cell morphology^40,41^. The observed nuclear elongation aligned to the orientation of the nanofibers suggests a cell morphology alignment. 53AH treatment in 3D induced a similar effect on nuclear morphology as the cells treated with XAV939 (Fig. S2 in Supplementary Information).

**Figure 4:**
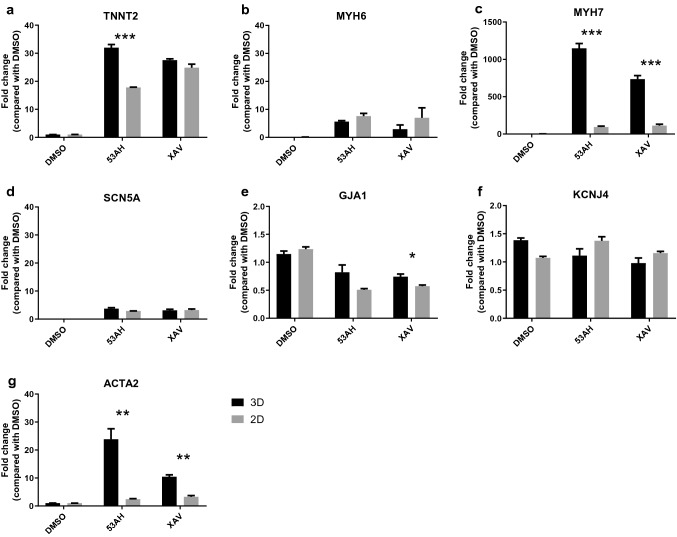
Real time RT-PCR analysis of CPC differentiation treated with Wnt inhibitors in 3D vs 2D culture. Human iPSC-CPCs were plated in 3D aligned nanofiber and 2D plates with the addition of 1.1 µM 53AH, 10 µM XAV939 and DMSO control from day 0 to day 3, followed by culture in assay medium until day 14 of differentiation. The expression of TNNT2, MYH6, MYH7, SCN5A, GJA1, KCNJ4, and ACTA2 were studied, with RPLP0 as a reference control. The relative expression levels of specific genes were calculated according to the formula 2^−ΔCt^, and normalized as fold change based on the corresponding DMSO controls. Results are presented as mean ± SEM. n = 3. *p < 0.05, **p < 0.01, ***p < 0.001 indicate significant difference between 3D and 2D cultures.

To further assess the effect of 3D nanofiber scaffolds on human iPSC-CPCs differentiation, cardiac and smooth muscle cell genes were analyzed. The RT-PCR results show that there is a higher expression of cardiac marker gene MYH7 in 53AH and XAV939 treated cells in 3D nanofiber plates compared with 2D plates at day 14 of differentiation. 53AH treatment also resulted in a higher expression level of TNNT2 in 3D nanofiber plates. However, 53AH and XAV939 treated cells show similar levels of SCN5A, MYH6, and KCNJ4 gene expression in 2D and 3D culture although these compound treated cells showed higher levels of SCN5A and MYH6 gene expression compared to DMSO vehicle treated cells. A slightly higher expression of GJA1 was observed in 3D culture compared to 2D culture. Moreover, a high expression of ACTA2 gene was observed in 53AH or XAV939 treated cells in 3D culture (Fig. 4).

**Figure 5:**
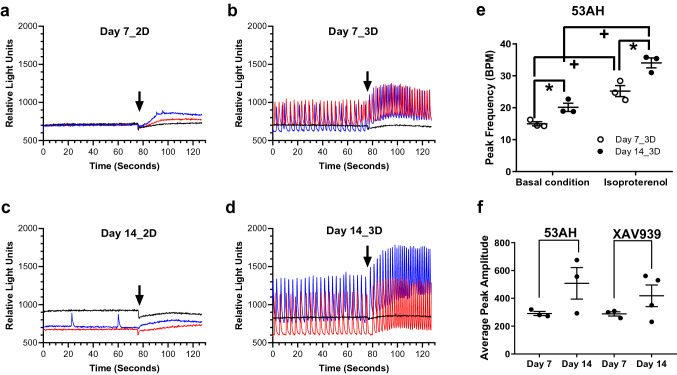
Effect of Wnt signaling inhibition on Ca^2+^ oscillation of CMs differentiated from CPCs in 3D vs 2D culture. Human iPSC-CPCs were plated in 3D aligned nanofiber and 2D plates with the addition of 53AH, XAV939 and DMSO control from day 0 to day 3, followed by culture in assay medium until day 7 or day 14 of differentiation. The cells were then loaded with FLIPR Calcium 5 dye and intracellular Ca^2+^ oscillation was recorded on a FLIPR Tetra system. (**a**–**d**) Representative recordings of intracellular Ca^2+^ oscillation from cells treated with 1.1 µM 53AH (blue tracers), 10 µM XAV939 (red tracers) or DMSO control (black tracers), and cultured in 2D plates for 7 days (**a**), 14 days (**b**) and in 3D aligned nanofiber plates for 7 days (**c**) and 14 days (**d**) with arrows indicating the time point of adding isoproterenol. (**e**,**f**) The peak frequency quantification of Ca^2+^ oscillation from cells differentiated with 53AH in 3D aligned nanofiber culture under the basal and isoproterenol stimulated conditions (**e**), and the peak amplitude of Ca^2+^ oscillation in the basal condition in cells treated with 53AH or XAV939 (**f**). Quantification results are presented as scatter dot plots with mean ± SEM. n = 3–4. *p < 0.05 indicates significant difference between Day 7_3D and Day 14_3D cultures. ^+^p < 0.05 indicates significant difference between the basal and isoproterenol stimulated conditions.

Contractile activity is one characteristic of functional CMs. Intracellular Ca^2+^ has been shown to be a surrogate for assessing CM contraction. In this study, treated cells were inspected under a microscope every day from day 5 post-compound treatment. Cells treated with XAV939 or 53AH in 3D nanofiber culture began displaying spontaneous contraction at day 6–7. At day 14, XAV939 and 53AH treated cells showed large beating areas in 3D culture, while only small beating clusters were observed in 2D culture. To further investigate the effect of 3D nanofiber scaffolds on CM function, we studied intracellular Ca^2+^ oscillation of differentiated cells in 3D and 2D culture using FLIPR Calcium 5 Ca^2+^ sensitive dye. 53AH or XAV939 treated cells in 3D nanofiber plates showed spontaneous synchronized Ca^2+^ oscillation at day 7 and 14. In comparison, no clear Ca^2+^ oscillation was observed in 2D culture of cells treated with 53AH or XAV939 at day 7 or 14. Representative intracellular Ca^2+^ recordings of cells treated with 53AH, XAV939 or DMSO in 3D versus 2D at day 7 and 14 are shown in Fig. 5a–d. CPC differentiated CMs in 3D nanofiber culture retained adrenergic responsiveness, as shown with increased Ca^2+^ oscillation rate induced by the addition of isoproterenol (Fig. 5c,d), which is confirmed by the quantification data showing an increased Ca^2+^ oscillation peak frequency at the isoproterenol stimulation condition as compared to the basal condition (Fig. 5e). The basal peak frequency in 53AH treated iPSC-CPCs in 3D nanofiber culture at day 14 was 20.1 beats per minute (BPM), which was slightly lower than the iPSC-CMs basal peak frequency, 24.2 BPM, with iPSC-CMs seeded directly on 3D aligned nanofiber plates and maintained for 10 days (Fig. S1). In addition, both 53AH and XAV939 treated cells in 3D showed a small increase in average peak amplitude (although not significant) was observed as the differentiation time increased from day 7 to 14 (Fig. 5f).

### Effect of BMP signaling inhibition and ALK5 inhibitor on human iPSC-CPC differentiation in 3D and 2D culture

**Figure 6:**
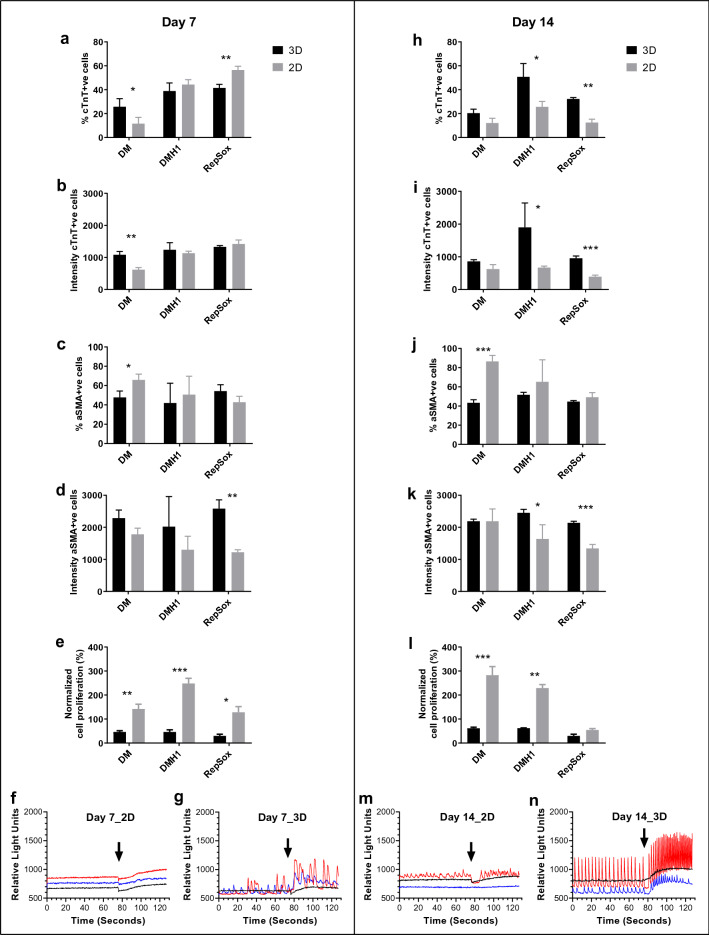
Effect of BMP signaling inhibition and ALK5 inhibitor on CPC differentiation in 3D vs 2D culture. Human iPSC-CPCs were plated in 3D aligned nanofiber and 2D plates with the addition of 1.1 µM DM, 1.1 µM DMH1 and 10 µM RepSox from day 0 to day 3, followed by culture in assay medium until day 7 or day 14 of differentiation. (**a**–**e**) At day 7 of differentiation in 2D and 3D cultures, % cTnT-positive cells, cTnT intensity of cTnT-positive cells, % of αSMA-positive cells, αSMA intensity of αSMA-positive cells, and effect on cell proliferation, (**f**–**g**) representative recordings of intracellular Ca^2+^ oscillation from cells treated with DM (blue tracers), DMH1 (red tracers) or RepSox (black tracers) in 2D and 3D culture for 7 days. Arrows indicate the time point of adding isoproterenol. (**h**–**n**) At day 14 of differentiation in 2D and 3D cultures, % cTnT-positive cells, cTnT intensity of cTnT-positive cells, % of αSMA-positive cells, αSMA intensity of αSMA-positive cells, effect on cell proliferation and intracellular Ca^2+^ oscillation. The effect on cell proliferation was normalized as the percentage of cell proliferation based on the on-plate DMSO controls. Results are presented as mean ± SEM. n = 3. *p < 0.05, **p < 0.01, ***p < 0.001 indicate significant differences between 3D and 2D cultures.

To further understand if 3D nanofiber culture in general is a better in vitro model for screening cardiac differentiation of human iPSC-CPCs, we tested DM and DMH1 (BMP signaling inhibitors) and RepSox (ALK5 inhibitor) that were identified to promote human iPSC-CPC cardiac differentiation using 2D culture^10^. DMH1 and RepSox treatments resulted in both a high percentage and high cTnT intensity of cTnT-positive cells in 3D culture compared to the 2D culture only at day 14. DM induced a higher percentage and high cTnT intensity of cTnT-positive cells in 3D at day 7, but the not at day 14 although there is still a similar trend (Fig. 6a,b,h,i). The percentage of αSMA-positive cells in 2D was higher than in 3D culture treated with DM at day 7 and 14 (Fig. 6c,j). αSMA-positive cells also showed higher αSMA intensity in 3D culture treated with DMH1 and RepSox at day 7 and 14. A high cell proliferation rate was observed in DM and DMH1 treated cells in 2D culture. In the FLIPR assay, DMH1 treated cells in 3D nanofiber plates showed spontaneously synchronized Ca^2+^ oscillation with an increased oscillation rate in response to isoproterenol at day 14 of differentiation, whereas, only small irregular Ca^2+^ oscillation was seen in 2D culture. At day 7, only irregular Ca^2+^ oscillation was observed in DMH1 treated cells. However, treatment with RepSox or DM induced either no Ca^2+^ oscillation or only Ca^2+^ oscillation with very small peak amplitudes in 3D and 2D cultures at day 7 and 14.

## Discussion

In vitro cellular models with cells grown in a condition that mimics the in vivo environment have the potential for improving translatability and providing better prediction of the effects of a compound in humans. Aligned nanofiber scaffolds, which structurally mimic the architecture of the cardiac ECM, are attractive model systems for studying cellular phenotypes including cell–cell interactions, proliferation, differentiation and functions. In this study, we investigated the effect of 3D aligned nanofiber scaffolds on cardiac differentiation of human iPSC-derived CPCs. Our results demonstrated that cells treated with inhibitors of Wnt signaling on 3D aligned nanofiber scaffolds showed: (1) improved CM differentiation of human iPSC-CPCs, as shown with increased number of cTnT-positive cells; (2) elongated nuclear morphology of αSMA-positive cells following the orientation of the aligned nanofibers; (3) reduced compound treatment induced cell proliferation; and (4) synchronized intracellular Ca^2+^ oscillation in cells.

Treating human iPSC-CPCs with Wnt inhibitors (53AH and XAV939) resulted in a significant increase in % cTnT-positive cells in 2D culture at day 14 of differentiation compared to cells treated with DMSO vehicle control. These results are consistent with previous reports demonstrating that the Wnt signaling pathway plays important roles in cardiac differentiation^10,39,42–45^.

Advantages of using 3D nanofiber scaffolds to support cell–cell interactions, direct cell alignment, and enhance CM differentiation of stem cells have previously been reported. It has been demonstrated that the expression of cardiac markers (e.g. cTnT and GATA4) were increased in 5-aza-2-deoxycytidine-treated human adipose-derived stem cells seeded on aligned or random nanofiber scaffolds compared to those in 2D cultures^37^. Chen and colleagues reported that 3D nanofibers combined with activation of Wnt/β-catenin signaling during the early period of cell differentiation could synergistically promote the CM differentiation of mouse iPSCs^46^. 3D culture conditions have also been shown to favor CM differentiation of ESC-derived and iPSC-derived cardiovascular progenitors^29^. These reports prompted us to investigate the effect of 3D culture on iPSC-CPC differentiation using 3D aligned nanofiber scaffolds which mimic the architecture of the native ECM of the heart. Our results show that the % cTnT-positive cells and cTnT intensity of cTnT-positive cells were higher in cells treated with the Wnt inhibitors 53AH or XAV939 on 3D aligned nanofiber scaffolds compared to 2D culture at day 7 or 14 of differentiation, demonstrating an improved CM differentiation in 3D aligned nanofiber culture. This is supported by the RT-PCR analyses showing that the expression levels of the CM marker genes TNNT2 and MYH7 were higher in 53AH treated cells, and MYH7 expression was higher in XAV939 treated cells seeded on 3D aligned nanofiber scaffolds compared with 2D culture. The increases in MYH7 and ACTA2 in the 3D groups may indicate a more ventricular-like phenotype, as MYH7 is predominantly expressed in fetal ventricles whereas MYH6 is found at higher levels in the atrium^47,48^ and ACTA2 expression has been shown to be enriched in ventricular-like cells in vitro^49^. Cell contraction is a characteristic of functional CMs, and synchronized Ca^2+^ oscillation has been shown to be a surrogate for assessing CM contraction. Furthermore, the FLIPR Ca^2+^ assay results and visual inspection show that the cells treated with the Wnt inhibitors and seeded on 3D aligned nanofiber scaffolds display spontaneous synchronized intracellular Ca^2+^ oscillation and cell contraction. These differentiated CMs also responded to adrenergic stimulation with increased Ca^2+^ oscillation frequency. However, in 2D culture, only small irregular or no synchronized Ca^2+^ oscillations were detected. The Ca^2+^ oscillation results are consistent with the immunofluorescence data showing a higher percentage and high cTnT intensity of cTnT-positive cells in 3D nanofiber culture treated with 53AH and XAV939 compared with 2D culture. These results demonstrate that human iPSC-CPCs treated with the inhibitors of Wnt signaling pathway in 3D nanofiber culture were found to be more committed to CM differentiation with synchronized Ca^2+^ oscillation and cell contraction than cells in 2D culture, and are in line with previous studies showing that simply manipulating the topography of the surface using 3D nanofiber scaffolds on which cells adhered to is able to improve CM differentiation. 3D aligned nanofiber scaffolds might increase cell-scaffold interactions via increased surface/volume ratio, which may contribute to the improved CM differentiation.

Adult CMs in the heart have an elongated morphology. Our nanofiber validation data demonstrate that human iPSC-CMs, seeded on 3D aligned nanofiber plates, show an elongated morphology that resembles native CMs in the heart, which is consistent with the results reported by several research groups^27,28^. These data suggest that aligned nanotopographic cues can guide CM cell alignment along the direction of fiber orientation and promote adaptation of an elongated CM morphology. Similar effects on cell morphology were reported for SMCs and fibroblastts^23,50,51^. However, CMs differentiated from iPSC-CPCs on 3D aligned nanofibers did not show cell alignment following the orientation of the nanofibers. The images taken at three planes from the confocal microscope showed that the cells located closer to the nanofibers were predisposed towards αSMA-positive cell differentiation and cells located further away from the nanofibers were predominately CMs. cTnT-positive CMs seemed not to be attaching to the fibers or penetrate into the spaces between the fibers, whereas the αSMA-positive cells were elongated in alignment with the fibers. The mechanism for the observed differences in αSMA-positive cells and cTnT-positive CMs location and morphology is unclear. Immature CMs and hESC-CMs cultured in vitro have been shown to express αSMA protein and ACTA2 gene^52,53^. Therefore, the observed αSMA-positive cells may represent a fraction of immature CMs, which can contribute to the improved Ca^2+^ oscillation and cell contraction as αSMA-positive CMs can show spontaneous Ca^2+^ oscillation and cell contraction. It would be interesting to study further if cardiac and smooth muscle cell maturation under the described conditions in 3D nanofiber culture is also contributing to the improved Ca^2+^ oscillation.

In a similar manner to our observations with the Wnt inhibitor treatment, the BMP inhibitor DMH1 was very effective at inducing CM differentiation in 3D culture, as shown with synchronized Ca^2+^ oscillation, high % cTnT-positive cells and high cTnT intensity of cTnT-positive cells in 3D culture, but only small/irregular Ca^2+^ oscillations and lower % cTnT positive cells as well as lower cTnT intensity in 2D culture at day 14. The DMH1 data further support the suggestion that 3D scaffolds can be a more relevant in vitro model system for studying CPC differentiation and for phenotypic screening of compounds inducing CPC differentiation to CMs as the observed improvement in 3D culture is not only limited for the Wnt signaling pathway inhibition. Another less selective BMP inhibitor DM was not very effective at inducing CM differentiation as it induced only 20% cTnT-positive cells and small/irregular Ca^2+^ oscillations in 3D culture. Furthermore, treatment with the ALK5 inhibitor RepSox did not induce synchronized Ca^2+^ oscillation either in 3D or in 2D culture although there were 41% and 56% cTnT positive cells, respectively, in 3D versus 2D culture at day 7. These results suggest that measuring only cardiac marker protein expression may not be enough for the identification of the most effective compounds and may lead to the selection of false positives, and highlight it is important to study and characterize compounds with a combination of marker protein measurement and functional Ca^2+^ oscillation readout of CMs. In addition, our results suggest the nanofiber scaffolds may play a role in the Wnt and BMP pathway regulation as human iPSC-CPCs treated with the inhibitors of the Wnt or BMP pathways in 3D nanofiber culture were found to be more committed to CM differentiation than cells in 2D culture. However, the mechanism for this is unclear and requires further investigation.

Many reports have indicated that 3D culture can affect cell proliferation. However, the effect of how 3D culture affects cell proliferation, as reported in the literature, is controversial. 3D culture was reported to induce a reduction in cell proliferation for cardiac fibroblasts within alginate scaffolds and for human adipose-derived stem cells on aligned and random PCL nanofiber scaffolds^15,54^. On the contrary, increased cell viability and proliferation was reported for human adipose-derived stem cells on aligned and random PCL nanofibrous scaffolds compared with 2D culture in a CM differentiation study of human adipose-derived stem cells. Our results are consistent with Dar and Brännmark et al. and showed that human iPSC-CPC proliferation was significantly diminished in the 3D aligned nanofiber culture together with improved cell differentiation. The theory that cell proliferation is in opposition to differentiation is supported by our results suggesting that CPCs need to be withdrawn from the cell cycle before cardiac differentiation and aligned nanofiber scaffolds may promote cardiac differentiation by switching off CPC proliferation.

In this study, we use a population of CPCs which are clearly defined and well described and characterized. However, a range of populations of CPCs can be obtained from different sources and genetic backgrounds after differentiation. As a next step it would be interesting to utilize the described nanofiber scaffolds and conditions to assess and compare the differentiation characteristics of these different CPC populations. In addition, the iPSC-CPCs used in this study is an enriched population of CPCs (characterized with 84.8% ± 3.4% KDR^pos^cKIT^neg^, 83.0% ± 2.7% KDR^pos^PDGFRα^pos^, and 76.5% ± 6.0% NKX2.5^pos^cTnT^neg^)^10^. A useful next step could be to further sort the CPCs of interest from the broader cell population to study the specific phenotype in closer detail. It will also be informative to increase the 3D nature of the nanofiber scaffolds further to see how far the phenotype can be enhanced.

## Conclusions

In summary, our study presents evidence that 3D aligned nanofiber scaffolds, with increased surface/volume ratio and aligned topography of the surface on which cells are adhered, improve iPSC-CPC differentiation to functional CMs across several means of inducing CM differentiation (Wnt and BMP inhibition) as shown with increased cTnT expression and synchronized intracellular Ca^2+^ oscillation. This work highlights the importance of using a more relevant in vitro model system and measuring not only the expression of CM marker proteins but also the functional readout of CMs in a screen in order to identify the best compounds and to investigate the resulting biology.

## Materials and methods

### Materials

Cryopreserved human iCell Cardiac Progenitor Cells (iPSC-CPCs) and human iCell Cardiomyocytes (iPSC-CMs) were purchased from Cellular Dynamics International (Madison, WI, USA) and stored in the vapour phase of liquid nitrogen until use. All chemical reagents (53AH, XAV939, Dorsomorphin (DM), Dorsomorphin homologue 1 (DMH1) and RepSox) were purchased from Sigma-Aldrich Sweden AB (Stockholm, Sweden) or synthesized at AstraZeneca unless stated otherwise. Cell culture medium and supplements were purchased from ThermoFisher Scientific (Stockholm, Sweden). FLIPR Calcium 5 Assay Kit was purchased from Molecular Devices (Berkshire, UK). RNA purification kits were purchased from Qiagen AB (Copenhagen, Denmark). TaqMan gene expression assays were purchased from ThermoFisher Scientific. 384-well and 96-well 2D Corning plates were purchased from VWR (Stockholm, Sweden) and 384-well and 96-well 3D aligned nanofiber plates were purchased from Nanofiber Solutions (Columbus, OH, USA). The thickness of the fiber layers is ~ 20 μm and the nanofiber polymers are approximate 700 nm in diameter (https://nanofibersolutions.com/).

### Cell culture and treatment

Cryopreserved human iPSC-CPCs (CPC-301-020-001-PT, Cellular Dynamics International) were thawed according to the manufacturer’s instructions and suspended in CPCs assay medium (William’s E medium supplemented with Cell Maintenance Cocktail B) (William’s E medium, A1217601, Cell Maintenance Cocktail B, CM4000, ThermoFisher Scientific). Cell number and cell viability were determined on a Cedex counter (Innovatis, Bielefeld, Germany). 2D black clear bottom 384-well plates (3,712/3,764, Corning) and 96-well plates (3,903, Corning), 3D 384-well aligned nanofiber plates (38,402, Nanofiber Solutions) and 96-well aligned nanofiber plates (9,602, Nanofiber Solutions) were coated with human fibronectin (1 µg/ml; Roche Applied Science) for at least 2 h at 37 °C. Compounds XAV939 (X3004, Sigma-Aldrich), 53AH, DM, DMH1 and RepSox were dissolved in dimethyl sulfoxide (DMSO) (D4540, Sigma-Aldrich) as 1,000 × stock solutions. For the 384-well imaging or FLIPR experiments, compounds were dosed in 50 nl/well using an Echo liquid handler (Labcyte, Sunnyvale, CA, USA) to fibronectin coated plates containing 25 µl/well of CPCs assay medium. DMSO was used as the vehicle control with a 0.1% final concentration. To compensate for the large surface area of 3D nanofiber plate compared to conventional 2D plates, a higher cell seeding density was initially used for the nanofiber plates. iPSC-CPCs were seeded to plates containing pre-dosed compounds at a density of 13,000 cells/well in 2D plates and 17,000 cells/well in 3D aligned nanofiber plates. For the 96-well gene expression experiments, compounds were dosed to fibronectin coated plates by adding 40 µl/well of 1/200 pre-diluted DMSO or compounds. iPSC-CPCs were seeded at 40,000 cells/well in 2D plates or 52,000 cells/well in 3D plates in 160 µl of CPCs assay medium. Cells with the addition of compounds from day 0 to day 3 were incubated at 37 °C with 5% CO_2_, followed by culture in fresh assay medium with medium refreshment every 48 or 72 h until the experiments were terminated at day 7 or day 14.

Cryopreserved human iPSC-CMs (CMC-100-110-001, Cellular Dynamics International) were thawed and suspended in iCell CM plating medium (CMM-100-110-005, Cellular Dynamics International) as described previously^55^. Cell number and viability were determined on a Cedex counter (Innovatis) and total cell number was determined with cell plating efficiency taken into account. Cells were seeded in 50 µl/well at 4,000 cells/well in gelatin-coated 384-well 2D Corning black clear bottom plates and at 6,000 cells/well in gelatin coated 384-well 3D aligned nanofiber plates and incubated at 37 °C for 48 h. The medium was then changed to iCell CM maintenance medium (CMM-100-120-005, Cellular Dynamics International) and maintained at 37 °C for 10 days with medium refreshment every 48 or 72 h.

### Immunofluorescence

Cells were fixed in 4% (w/v) formaldehyde (dilute 37% formaldehyde solution with PBS, F8775, Sigma-Aldrich) on the indicated day for 20 min at room temperature (RT), permeabilized with 0.1% Triton X-100 in PBS for 15 min and then blocked in 5% FBS/TPBS block buffer (with 0.02% Tween-20 in PBS) for 30 min. The cells from the CPCs differentiation study were then stained with primary antibodies [rabbit anti-Cardiac Troponin T (cTnT) (1:400, ab45932, Abcam), mouse anti-human smooth muscle actin (αSMA) (1:100, Clone 1A4, M0851, Dako)] overnight at 4 °C. After overnight incubation, cells were washed with PBS followed by 45 min incubation with secondary antibodies Alexa Fluor 647 Donkey anti-rabbit IgG (H + L) (1:500, A-31573, ThermoFisher Scientific), Alexa Fluor 488 Goat anti-mouse IgG (H + L) (1:500, A-11001, ThermoFisher Scientific) and 20 min with Hoechst 33342 (62249, ThermoFisher Scientific) at RT followed by final washing with PBS. The iPSC-CMs were stained with primary antibody rabbit anti-cTnT followed by secondary antibody Alexa Fluor 488 Goat anti-rabbit IgG (H + L) (1:500, A-11034, ThermoFisher Scientific) and Hoechst 33342. The plates were sealed and imaged on an ImageXpress XL system (Molecular Devices, Berkshire, UK) using 20 × (4 sites per well) or 4 × (one site per well) objectives. The plates with 14-day differentiation were also imaged on a Yokogawa CV7000 confocal microscope (Wako automation, San Diego, California, USA) using a 20 × air objective and 6 µm intervals. Images were analyzed using Columbus image analysis software (PerkinElmer).

### Real-time reverse transcription polymerase chain reaction (RT-PCR)

For analysis of the gene expression, treated and untreated cells were washed with PBS and lysed using RNeasy cell lysis buffer RLT (RNeasy Mini kit, 74104, Qiagen, Copenhagen, Denmark) to obtain 350 µl cell lysate/sample. The total RNA was purified with RNeasy Mini kit using RNA Cell Method with DNase digest protocol and 30 µl elution volume on a robotic RNA purification system QIAcube (Qiagen) following the instructions from the manufacturer. Purified RNA samples were stored at − 80 °C until analysis. mRNA was reverse transcribed to cDNA using random primers and High capacity cDNA reverse transcription kit (4368813, Applied Biosystems, Foster City, CA, USA). Real time RT-PCR was conducted with Taqman technology to assess gene expression of the selected human genes, TNNT2 (cardiac troponin T, Hs00165960_m1, ThermoFisher Scientific), MYH6 (myosin heavy chain 6, Hs01101425_m1, ThermoFisher Scientific), MYH7 (myosin heavy chain beta, Hs01110632_m1, ThermoFisher Scientific), SCN5A (Nav1.5, Hs00165693_m1, ThermoFisher Scientific), KCNJ4 (inward rectifier potassium channel 4, Hs00705379_s1, ThermoFisher Scientific), GJA1 (gap junction protein alpha 1, Hs00748445_s1, ThermoFisher Scientific), and ACTA2 (alpha smooth muscle actin, Hs00426835_g1, ThermoFisher Scientific). Ribosomal Protein Large P0 (RPLP0), using forward primer 5′–3′ CCATTCTATCATCAACGGGTACA, reverse primer 5′–3′ AGCAAGTGGGAAGGTGTAATCC and probe 5′ FAM-TCTCCACAGACAAGGCCAGGACTCGT-TAMRA 3′ (Sigma-Aldrich), was used as endogenous control. Fourty cycles of reactions were performed in triplicates for each sample on an Applied Biosystems QuantStudio 7 instrument (ThermoFisher Scientific). Only samples reaching threshold values before cycle 36 were included. The relative expression levels of specific genes were calculated using the formula 2^−dCt^, where dCt is the difference in threshold cycle (Ct) values between the target gene and the endogenous control gene RPLP0.

### Intracellular calcium oscillation

At day 7 or day 14 of iPSC-CPCs differentiation, or at day 10 of iPSC-CMs maintenance, intracellular Ca^2+^ oscillation was studied on a FLIPR Tetra system (Molecular Devices, Berkshire, UK) using FLIPR Calcium 5 Assay Kit (Molecular Devices) as described by Pointon et al.^55^. Briefly, the cell culture media were first removed and replaced with 25 µl/well of fresh culture medium 2 h prior to assay. The cells were loaded with 1 × FLIPR Calcium 5 dye at 37 °C for 40 min by adding 25 µl/well of 2 × FLIPR Calcium 5 dye. The cell plates were transferred to a FLIPR Tetra system and maintained at 37 °C. Ca^2+^ oscillations were recorded at Ex 485 nm/Em 530 nm with 0.05 s exposure time/read and 0.12 s read interval. 450 reads were collected at the basal condition, followed by a further 350 reads post-the addition of 12.5 µl/well of 10 µM Isoproterenol solution (prepared from 10 mM stock, I5627, Sigma-Aldrich, diluted in assay medium) to reach a final concentration of 2 µM isoproterenol using onboard liquid handling within the FLIPR Tetra, allowing both pre- and post- compound reads to be obtained from the same well from plates maintained at 37 °C during the recording period. Ca^2+^ oscillation peak frequencies and peak amplitude were analyzed using the ScreenWorks Peak Pro Software (Molecular Devices).

### Statistical analysis

Data are presented as mean ± SEM (n = 3). Statistical analysis was performed with multiple *t* tests correct for multiple comparisons using the Holm-Sidak method in GraphPad Prism 7 (GraphPad Software, California, USA). Value of p < 0.05 was considered to be significant and was indicated with *p < 0.05, **p < 0.01 and ***p < 0.001.

## Supplementary information

Supplementary Figures.
