# Supplementary material for: Aligned nanofiber scaffolds improve functionality of cardiomyocytes differentiated from human induced pluripotent stem cell-derived cardiac progenitor cells
Source: Sci Rep. 2020 Aug 11;10:13575. doi: 10.1038/s41598-020-70547-4 (PMC7419298; doi:10.1038/s41598-020-70547-4)

**Aligned nanofiber scaffolds improve functionality of cardiomyocytes differentiated from human induced pluripotent stem cell-derived cardiac progenitor cells**

Mei Ding^1*¶^, Henrik Andersson^1¶^, Sofia Martinsson^2^, Alan Sabirsh^3^, Anna Jonebring^1^, Qing-Dong Wang^2^, Alleyn T. Plowright^4^, Lauren Drowley^2^

^1^ Discovery Biology, Discovery Sciences, R&D, AstraZeneca, Gothenburg, Sweden

^2^ Bioscience Cardiovascular, Research and Early Development, Cardiovascular, Renal and Metabolism (CVRM), BioPharmaceuticals R&D, AstraZeneca, Gothenburg, Sweden

^3^ Advanced Drug Delivery, Pharmaceutical Sciences, R&D, AstraZeneca, Gothenburg, Sweden

^4^ Medicinal Chemistry, Research and Early Development, Cardiovascular, Renal and Metabolism (CVRM), BioPharmaceuticals R&D, AstraZeneca, Gothenburg, Sweden

* Corresponding author

E-mail: [mei.ding@astrazeneca.com](mailto:mei.ding@astrazeneca.com) (M.D.)

^¶^ These authors contributed equally to this work

**Supplementary Information**

**Figure S1: Immunofluorescence staining and measurement of intracellular Ca^2+^ oscillation of human iPSC-Cardiomyocytes (iPSC-CMs) seeded in 3D aligned nanofiber and 2D standard culture plates**. Human iPSC-CMs were plated in 3D aligned nanofiber and 2D plates and cultured for 10 days. Cells were either fixed and stained for cTnT and Hoechst, and imaged at 20X magnification, or loaded with FLIPR Calcium 5 dye for the measurement of cardiac contraction as described in the methods. Human iPSC-CMs, seeded on 3D aligned nanofiber plates, showed an elongated morphology (B) that resembles native CMs in the heart, whereas CMs cultured in 2D plates showed a non-oriented morphology (A). (C-D) Representative recordings of spontaneous Ca^2+^ oscillations from iPSC-CMs seeded in 2D culture (C) and 3D culture (D) with empty arrows indicating the time point of adding isoproterenol. (E-F) The peak frequency quantification of Ca^2+^ oscillation from iPSC-CMs cultured in 3D nanofiber and 2D cultures under the basal and isoproterenol stimulated conditions (E), and the peak amplitude of Ca^2+^ oscillation in the basal condition (F). Quantification results are presented as scatter dot plots with mean ± SEM. n=3. *** P<0.001 indicates significant difference between 3D and 2D cultures.


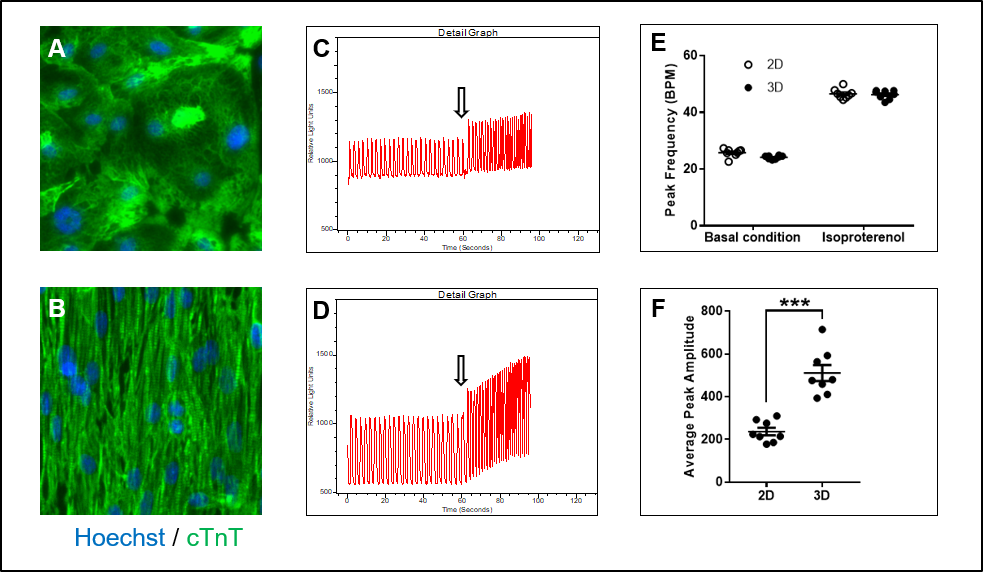


**Figure S2. Confocal microscopy of CPC differentiation treated with 53AH in 3D vs 2D culture** Human iPSC-CPCs were plated in 3D aligned nanofiber and 2D plates with the addition of 1.1µM 53AH from day 0 to day 3, followed by culture in assay medium until day 14 of differentiation. Hoechst staining (grey color), and expression of cTnT (red color) and αSMA (green color) were studied by acquiring images at 3 z-planes using 20X air objective at 6µm intervals. Plane 1 is close to the plate bottom level, Plane 2 is 6 µm above plane 1, and Plane 3 is 12 µm above plane 1. (a-f) Representative images of cells treated with 10 µM XAV939. Cells located closer to the fibers in 3D aligned nanofiber culture displayed elongated nuclei aligned to the orientation of the nanofibers (plane 1 in panel b). cTnT-positive CMs (panel d) were found to be located relatively distant from the fibers compared with αSMA-positive cells (panels f).


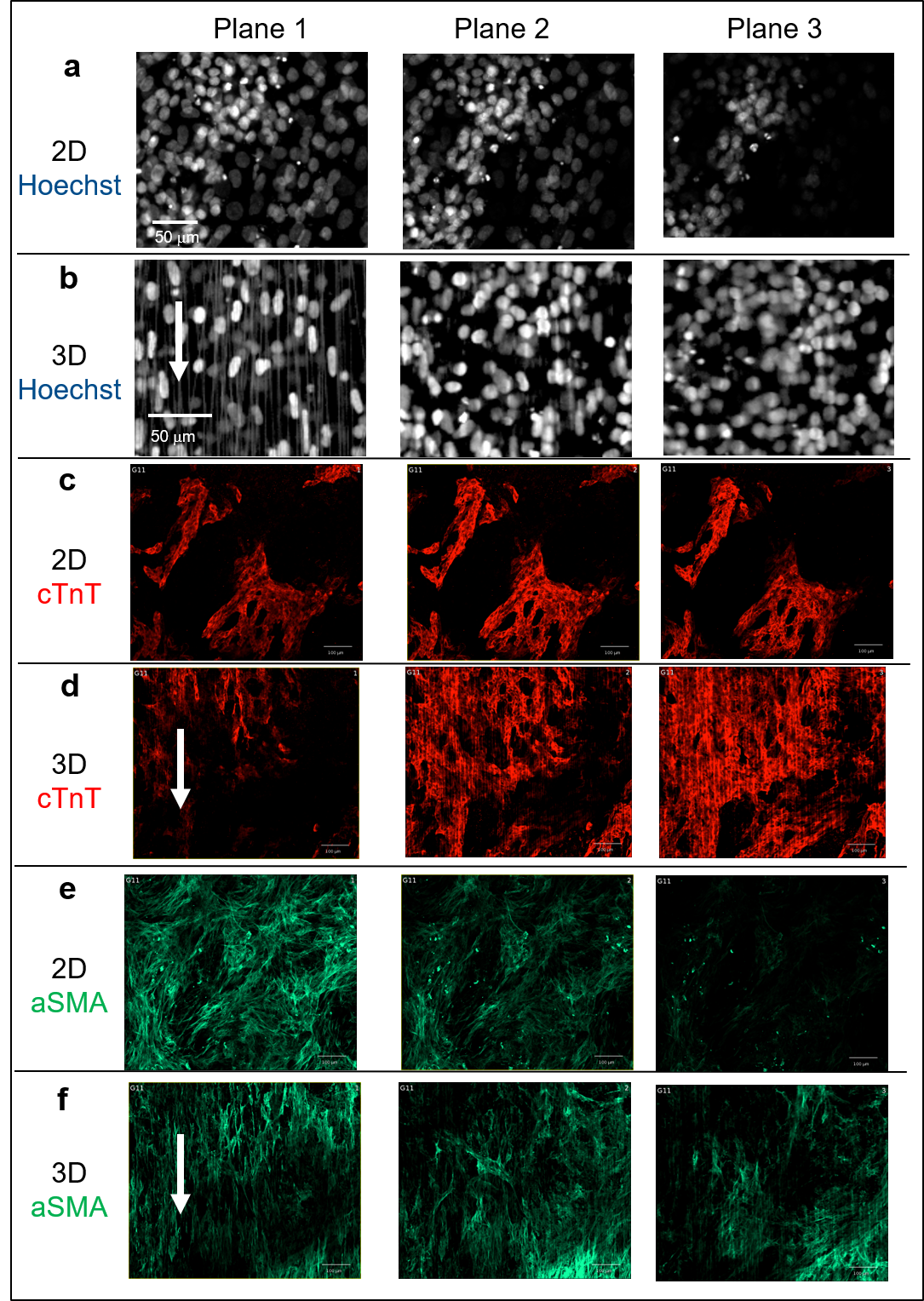

Supplement: Supplementary file 1 — Supplementary Figures. [file 41598_2020_70547_MOESM1_ESM.docx]
